# Twelve-month outcomes and comparative costs of internet-delivered psychodynamic therapy versus cognitive-behavioral therapy for adolescent depression: a randomized controlled trial

**DOI:** 10.3389/fpsyt.2026.1794684

**Published:** 2026-04-20

**Authors:** Karin Lindqvist, Jakob Mechler, Paraskevi Peristera, Per Carlbring, Fredrik Falkenström, Peter Lilliengren, Gerhard Andersson, Robert Johansson, Nick Midgley, Julian Edbrooke-Childs, Hanne-Sofie J. Dahl, Rolf Sandell, Agneta Thorén, Naira Topooco, Randi Ulberg, Katja Lindert Bergsten, Björn Philips

**Affiliations:** 1 Department of Psychology, Stockholm University, Stockholm, Sweden; 2 The Erica Foundation, Stockholm, Sweden; 3 Department of Psychology, Uppsala University, Uppsala, Sweden; 4 School of Psychology, Korea University, Seoul, Republic of Korea; 5 Department of Psychology, Linneaus University, Växjö, Sweden; 6 Department of Behavioural Sciences and Learning, Linköping University, Linköping, Sweden; 7 HEI-Lab: Digital Human-Environment Interaction Labs, Lusófona University, Lisboa, Portugal; 8 Anna Freud/University College London, London, United Kingdom; 9 Department of Psychology, University of Oslo, Oslo, Norway; 10 Department of Psychology, Lund University, Lund, Sweden; 11 Division of Mental Health and Addiction, University of Oslo and Oslo University Hospital, Oslo, Norway

**Keywords:** adolescent depression, CBT, cost-analysis, follow-up, internet-delivered treatment, PDT, RCT

## Abstract

**Introduction:**

Adolescent depression poses a major public health concern with substantial clinical and societal implications. Both internet-delivered cognitive behavioural therapy (ICBT) and internet-delivered psychodynamic therapy (IPDT) have shown efficacy, but questions remain regarding long-term efficacy and cost-effectiveness. The present study presents a 12-month follow-up and cost-comparison from a randomized controlled trial (RCT) comparing ICBT and IPDT for adolescent depression.

**Methods:**

Participants were 272 adolescents aged 15–19 with a primary diagnosis of major depressive disorder. The primary outcome was depressive symptoms measured with the QIDS-A17-SR while the secondary outcome was anxiety symptoms measured with the GAD-7. Costs were assessed both by comparing costs of treatment and healthcare use 12-month post-treatment using the TIC-P.

**Results:**

Results were stable at the 12-month follow up compared to treatment endpoint, for both depressive and anxiety symptoms. There were no significant group differences at the 12-month follow-up. There were no differences in treatment costs or in costs for healthcare use one-year post-treatment.

**Discussion:**

This study suggests that treatment gains from IPDT and ICBT for adolescent depression remain stable during a 12-month follow-up period, with no differences between the treatments one-year post-treatment. Furthermore, it suggests comparable costs for the treatments. Interpretation of health-care use data was restricted due to the COVID-19 pandemic taking place during the follow-up period. This adds to the literature suggesting that ICBT and IPDT can be seen as viable alternatives for treating adolescent depression. More research into the long-term effects and cost-effectiveness is needed.

## Introduction

Adolescent depression represents a major public health concern with substantial clinical implications. According to the World Health Organization, it ranks among the leading causes of illness and disability in youth worldwide and is associated with considerable functional impairment, academic underachievement, and increased risk for suicidality. Given its prevalence, long-term course, and impact on developmental trajectories, adolescent depression remains a critical focus for clinical research and intervention efforts (1).

With adolescent depression prevalence currently on the rise, it is worrying that many are not receiving adequate treatment (2–4). In general, adolescents with depression seem to respond less favourably to antidepressant medication compared to depressed adults (5). Recent research has highlighted the fact that the efficacy of fluoxetine for paediatric depression has declined to clinically unimportant levels over time. This perceived decline in efficacy likely reflects the accumulation of more rigorous trial data and updated systematic reviews which suggest that earlier effects may have been over-estimated, yet these findings remain largely unacknowledged in clinical guidelines (6). Psychological treatments based on different theoretical frameworks have been developed and evaluated for adolescent depression (e.g., 7–9). Overall, Cuijpers et al. (10) found that 39% of children and adolescents receiving some type of psychotherapy treatment achieved a clinically significant improvement at treatment termination. However, access to psychiatric care may be limited by several factors, such as logistical, financial, or geographic barriers. Furthermore, young people may avoid seeking healthcare due to perceived stigma and/or a wish to be more self-reliant (11). This points to the need for accessible healthcare alternatives for a young population that are in dire need of low-threshold treatments.

In recent years, the increase of internet-delivered psychological interventions - notably cognitive behavioural therapy (ICBT) and psychodynamic therapy (IPDT) - has drastically expanded access to low-threshold, evidence-based care (11). Meta-analytic results indicate that ICBT leads to long-term positive effects on psychological disorders (12) including for adult depression (13). Furthermore, evidence indicates that ICBT can yield outcomes comparable to those of conventional face-to-face therapy in adults, while offering additional advantages in terms of accessibility and flexibility (14). Similarly, recent developments have enabled the adaptation of psychodynamic psychotherapy for guided online self-help formats, leading to growing empirical support for IPDT’s utility in treating mood and anxiety disorders (e.g., 15–20). Previous trials on IPDT for adult depression and anxiety have shown maintained, and sometimes improved, effects on follow-ups ranging from 6–23 months (15, 16, 19, 21). Digitally delivered interventions have also been tested for children and adolescents. Although evidence is slowly accumulating, the evidence-base is more limited compared to adult populations (22). In the treatment of adolescent depression, a recent meta-analysis found ICBT to be effective compared to control conditions (SMD = 0.42; 23) and IPDT has shown to be effective compared to control conditions (24) and non-inferior compared to ICBT (18). Even more uncertainty concerns the long-term effects of internet-delivered treatment for adolescent depression. Given the high rates of relapse and the chronic nature of depressive episodes during development (e.g., 25), understanding whether treatment gains persist beyond the immediate post-intervention phase is critical for determining the true clinical value of these modalities (26). Therefore, researchers underscore the need not only to examine improvements at post-treatment, but also to evaluate long-term outcomes and the economic implications of interventions (23, 27).

From a societal perspective, the cost-effectiveness of interventions is critical, as it informs decisions on resource allocation that aim to optimize overall well-being and social welfare. Analyses of cost-effectiveness may encompass direct healthcare costs as well as indirect societal cost, such as those related to disability, productivity loss due to inability to work, and need for informal caregiving. For children and adolescents, indirect societal costs differ from those observed in adults, as most do not participate in the labor market and therefore do not incur productivity losses themselves. However, mental health problems in this age group may be associated with indirect costs borne by parents or caregivers, such as reduced work productivity or increased caregiving demands. Consequently, societal costs related to child and adolescent mental health may include factors such as delinquency and informal caregiving, although it has been recommended that economic evaluations restrict their analyses to direct medical costs only (e.g., 28). A large comparative trial showed no significant differences in costs between PDT and CBT delivered face to face for adolescent depression (29). Internet-delivered interventions are often described as a cost-effective choice (30), however, this is far from always assessed in studies. Meta-analytic results indicate that internet-delivered interventions are cost-effective for mental disorders, but with mixed results (31). More recent meta-analytic data suggests that guided internet interventions are likely cost-effective compared to usual care, as the clinical gains from therapist support often justify the additional delivery costs (27, 32).

The present study addresses existing gaps in the literature by evaluating long-term clinical outcomes up to 12 months and comparing treatment and healthcare costs of internet-delivered CBT and PDT for adolescent depression. The study comprises a follow-up and cost-analysis on data from an RCT. Main outcomes have been published, indicating efficacy for both treatments at post-treatment with no differences in primary or secondary outcomes (18). The primary objective of the study was to assess maintenance of depressive symptom improvement during the 12-month follow-up, as well as between-group differences in trajectories during this time. Secondary objectives were maintenance of anxiety symptom improvement, as well as an economical evaluation encompassing a comparison of a) delivery costs and b) direct medical costs for the healthcare system during follow-up.

## Methods

This is a follow-up and cost-comparison of an RCT comparing ICBT and IPDT for adolescent depression (18), registered at ISRCTN12552584. The study was approved by the Swedish Ethical Review Authority on Aug 14, 2019 (reference number 2019-03023). Recruitment was conducted nation-wide in Sweden between August 2019 and October 2020. During this time, 272 adolescents were randomized (1:1) to one of the two conditions. Randomization was conducted using permuted block randomisation (1:1) by two independent researchers with no other involvement in the study. Randomisation was conducted after final enrolment and completion of all baseline measures. Inclusion criteria comprised a diagnosis of major depressive disorder (MDD), defined by a score of ≥9 on the Quick Inventory of Depressive Symptomatology – Adolescent self-rated version (QIDS-A17-SR; 33) and confirmation using the Mini International Neuropsychiatric Interview (MINI 7.0; 34), age between 15 and 19 years, and sufficient proficiency in Swedish. Exclusion criteria included substantial risk of suicide (i.e., clear intent or plans) or earlier suicide attempts, psychotropic medication not stable in the past month (or with planned adjustments within the coming 3 months), ongoing participation in other psychological treatment(s), or inability to comprehend what it meant to participate in the research. A primary diagnosis other than MDD, as well as any psychotic disorder, bipolar disorder, antisocial personality disorder, alcohol or substance use disorder, or autism spectrum disorder were also causes for exclusion.

### Participants

A total of 272 participants were included. Mean age was 17.32 (SD = 1.27) and 83% identified as female. Psychiatric diagnoses according to the MINI 7.0 are presented in **Table 1**. Results from the treatment period are presented in Mechler et al. (18). In short, both treatment groups improved significantly, with no differences in efficacy between IPDT and ICBT after 10 weeks of treatment.

**Table 1:** Diagnoses at baseline, according to MINI 7.0.

Diagnosis, n (%)	ICBT	IPDT
Major depressive disorder, recurrent	93 (68)	101 (74)
Persistent depressive disorder (≥ 1 year)	47 (35)	58 (43)
Panic disorder	21 (15)	13 (10)
Agoraphobia	18 (13)	11 (8)
Social anxiety disorder	48 (35)	32 (24)
Generalized anxiety disorder	35 (26)	35 (26)
Posttraumatic stress disorder	11 (8)	16 (12)
Obsessive compulsive disorder	7 (5)	6 (4)
Bulimia Nervosa	4 (3)	6 (4)
Binge eating disorder	2 (1)	5 (4)
Nonsuicidal Self-Injury, current	18 (13)	18 (13)
Nonsuicidal Self-Injury, past	33 (24)	42 (31)

### Interventions

Both interventions were 10-week guided self-help interventions delivered on a secure platform designed for the purpose of delivering digital interventions (35). Conditions were matched on format as well as mode of and frequency of guidance, following a handbook developed specifically for the project. In both conditions, participants received a weekly module for the first eight weeks, consisting of text, videos, audio, and exercises. Each participant had a therapist who responded to exercises and messages within approximately 24 hours on weekdays. In addition, participants were offered weekly synchronous chat sessions of 30 minutes. The treatment programs have been described in detail in other papers (36–38). Therapists were clinical psychologist students in their final year of training. All therapists received a full day of training in their respective methods and 120 minutes of weekly expert group supervision.

### Instrumentation

The primary outcome measure was the QIDS-A17-SR (33). This choice of instrument is particularly relevant given recent evidence suggesting that more commonly used measures, such as the Patient Health Questionnaire (PHQ-9; 39), may lack the temporal and group measurement invariance necessary to accurately monitor clinical change across treatment weeks (40). The QIDS-A17-SR has demonstrated reliability and validity in adolescent samples and covers all symptoms of depression according to the DSM-5, including the irritability criterion (33).

The secondary outcome measure used in the long-term follow-up was the Generalized Anxiety Disorder– 7 (GAD-7; 41). The GAD-7 is widely used to assess symptoms of anxiety as it is a valid, reliable, and brief measure, hence suitable for frequent assessments. Like QIDS-A17-SR, GAD-7 has been found to be valid and reliable also in adolescent populations (42).

For the estimations of treatment costs, we used the time reported by therapists to calculate time spent on messages and response to exercises, as well as infrequent phone calls (mainly as a safety procedure in the case of worry about a participant, for example for expressing suicidal thoughts). Time spent in chat sessions were logged automatically. The time used was multiplied with the calculated costs for a psychologist. Here, we used the mean value between a psychologist in primary care and in specialized care. It should be noted that both treatments are designed to be exactly equally time consuming, and any differences in time spent by psychologist are therefore due to patient engagement and occasional deviations by therapists from the prescribed time allocation. Therapist time distribution is described in **Supplementary Table 1**.

For the estimation of direct medical care costs, we used the Trimbos and Institute of Medical Technology Assessment Cost Questionnaire for Psychiatry (TIC-P; 43). Following recommendations for the use of TIC-P with adolescents, only the sections concerning direct medical care costs were used (28). Items in TIC-P include visits to different categories of health care, both somatic and psychiatric, and both out- and inpatient care, as well as emergency care. Health care visits were then multiplied with the costs for the respective health care personnel, based on data from The Swedish Association of Local Authorities and Regions (Sveriges Kommuner och Regioner). Unit costs and mean units are described in **Supplementary Table 2**. When describing costs, we converted from SEK to USD using the mean conversion rate for 2021, 0.1166, reflecting the price level at the time of study treatment implementation.

For the present study, all questionnaires were administered at endpoint, as well as 1, 6 and 12-months post-treatment, except for the TIC-P for which the pre-treatment measurement and the 12-month post-treatment measurement were used.

### Statistical methods

Long term effects on symptoms of depression and anxiety were analysed using linear mixed growth models. Follow-up time was coded with end of treatment as 0, and 1, 6 and 12 representing months after treatment termination. To model the trajectory of change over time, we compared several functional forms of the time variable, including linear, quadratic, and unstructured (categorical) time trends. Model fit was assessed using AIC, with reductions of ≥2 indicating a better fitting model (44). An unstructured variance–covariance matrix was specified for the random effects, placing no constraints on the variances or covariances and allowing for correlation between the random intercept and the random slope for time. The models contained fixed effects for time, group and the interaction between time and group. For QIDS-A17-SR, the best fitting model was a linear model with random intercept and slope. For GAD-7, the best fitting model was a model with unstructured time, and random intercept and slope. All models were estimated using restricted maximum likelihood (REML). Analyses were performed using Stata 18.5.

For costs, descriptive analyses of pre- and post-intervention healthcare costs were first calculated for each treatment group separately, reporting both mean (SD) and median (IQR) values. These analyses were intended to provide context and illustrate changes over time but were not used for statistical inference regarding treatment effects. As differences in clinical effect between the treatment groups were small, calculating an incremental cost-effectiveness ratio (ICER) could have led to misleading or inflated estimates (45). Therefore, no ICER was calculated. Instead, we compared healthcare costs at 12-month follow-up between groups using a generalized linear model (GLM) with a gamma distribution and log link, which is appropriate for positively skewed cost data (46, 47). Baseline (pre-intervention) healthcare costs were included as a covariate to adjust for pre-existing differences and improve precision. Results are presented as adjusted mean costs per group and the absolute difference between groups, where adjusted means represent model-based expected costs standardized to the same baseline cost distribution across treatment groups.

All zero costs were retained in the GLM, as the log link can accommodate small positive values.

Extreme cost values were addressed using winsorization as part of sensitivity analyses, with costs below the 1st percentile replaced by the 1st percentile value and costs above the 99th percentile replaced by the 99th percentile value.

Missing data was handled under the assumption that data was missing at random (MAR). Patients with missing follow-up cost data were included in the GLM using available-case analysis, which is valid under the MAR assumption. Sensitivity analyses, including winsorization of extreme cost values and bootstrap estimation of confidence intervals, were performed to assess the robustness of the findings.

## Results

Questionnaire completion rates across the various time points and measures ranged from 86.8% to 96%. Exact completion frequencies for each measure and timepoint are presented in **Table 2**.

**Table 2:** Estimated mean values from linear mixed model analysis.

Measure	Condition	Time	Estimate	SE	95% CI	n
QIDS-A17-SR	ICBT					
		Post-treatment	9.01	0.48	8.06, 9.96	132
		Follow-up 1m	9.04	0.47	8.13, 9.96	122
		Follow-up 6 m	9.20	0.44	8.33, 10.06	123
		Follow-up 12 m	9.39	0.52	8.37, 10.41	122
	IPDT					
		Post-treatment	8.91	0.49	7.96, 9.86	129
		Follow-up 1 m	9.00	0.47	8.08, 9.92	119
		Follow-up 6 m	9.45	0.45	8.58, 10.33	114
		Follow-up 12 m	9.99	0.53	8.94, 11.04	114
GAD-7	ICBT					
		Post-treatment	7.92	0.45	7.04, 8.81	130
		Follow-up 1 m	7.03	0.45	6.14, 7.93	121
		Follow-up 6 m	7.43	0.46	6.53, 8.32	123
		Follow-up 12 m	7.01	0.51	6.01, 8.01	122
	IPDT					
		Post-treatment	7.23	0.46	6.34, 8.13	125
		Follow-up 1 m	6.77	0.46	5.87, 7.68	118
		Follow-up 6 m	7.38	0.47	6.46, 8.30	113
		Follow-up 12 m	7.59	0.52	6.57, 8.62	114

### Concurrent treatment for depression during follow-up

At the 12-month follow-up, participants were asked if they had received any treatment for depression since their study completion. In the ICBT group, 25 participants (18%) reported starting some kind of talking treatment for depression. For the IPDT group, the corresponding number was 26 (19%). Two participants (1%) in the ICBT group, and five (4%) participants in the IPDT group reported already having started another talking treatment at the time of the study completion and having continued that. Regarding pharmacological treatment for depression, 17 participants (13%) in the ICBT group and 19 participants (14%) in the IPDT group reported having initiated medication after study completion. Seven participants (5%) in the ICBT group reported already being on medication at the time of study completion and having continued that, while the corresponding number for IPDT was six (4%). For the group stating that they had been in talking treatment for depression since completion of the study, the mean number of sessions reported was 13.67 (SD 13.22) for ICBT participants and 13.80 (SD 10.87) for IPDT participants. For the entire group, the mean number of sessions was 2.71 (SD 7.98) for the ICBT group and 3.04 (SD 7.64) for the IPDT group.

### Long-term follow-up

#### QIDS-A17-SR

**Figure 1:**
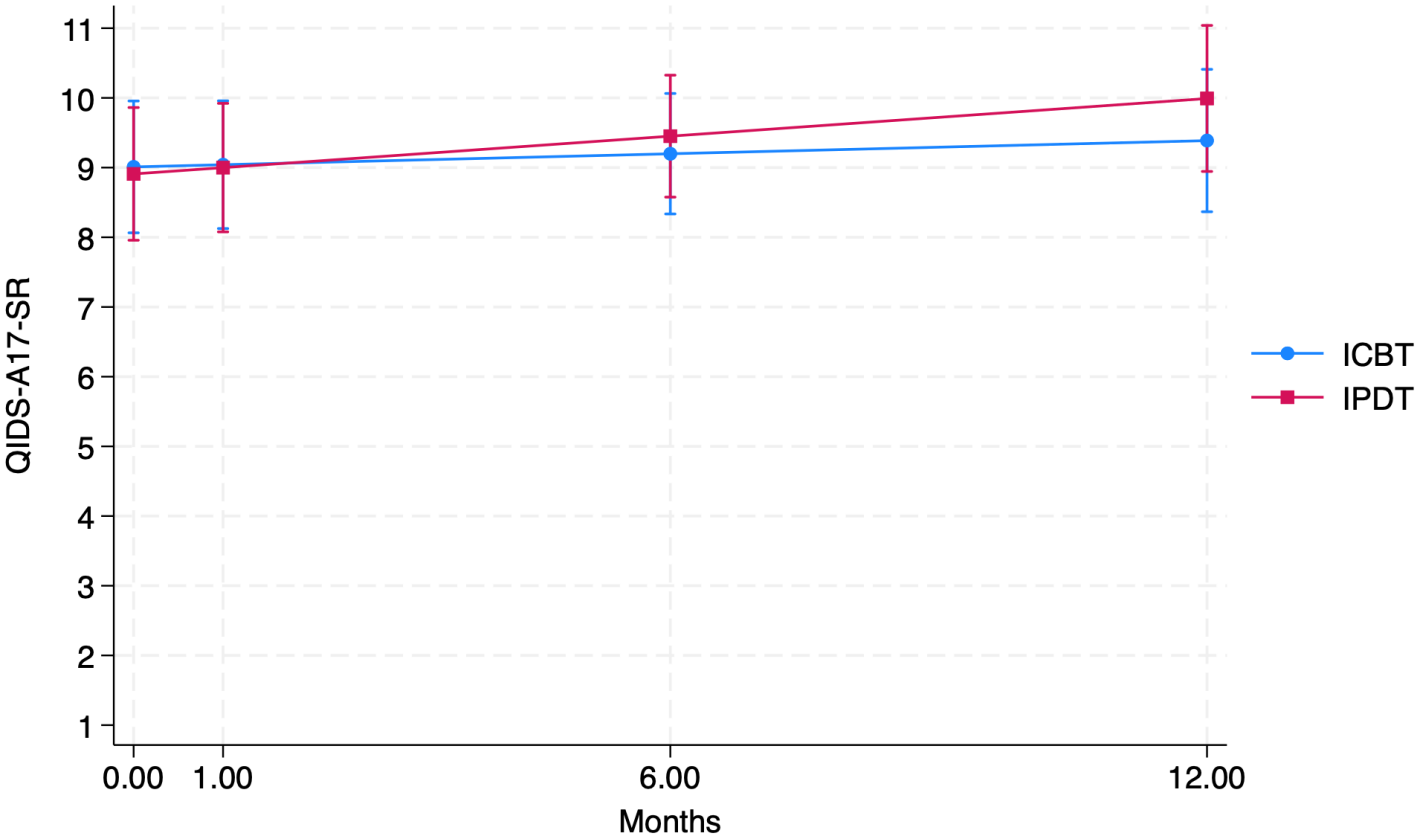
Estimated QIDS-A17-SR scores at post-treatment, 1m, 6m and 12m follow-up.

There were no significant fixed effects of either time (*p =* 0.431) or the interaction between time and group (*p* = 0.308), suggesting that changes were stable with no group differences in slope during the follow-up time. Average depressive symptom levels were within the mild range (33). The non-significant difference in estimated change between groups in raw scores was -0.7 points (95% CI -2.06, 0.65), corresponding to an effect size of *d* = -0.21 (95% CI -0.61, 0.19). See **Table 2** for estimated means for each time-point, and **Figure 1** for an illustration of the trajectories.

#### GAD-7

**Figure 2:**
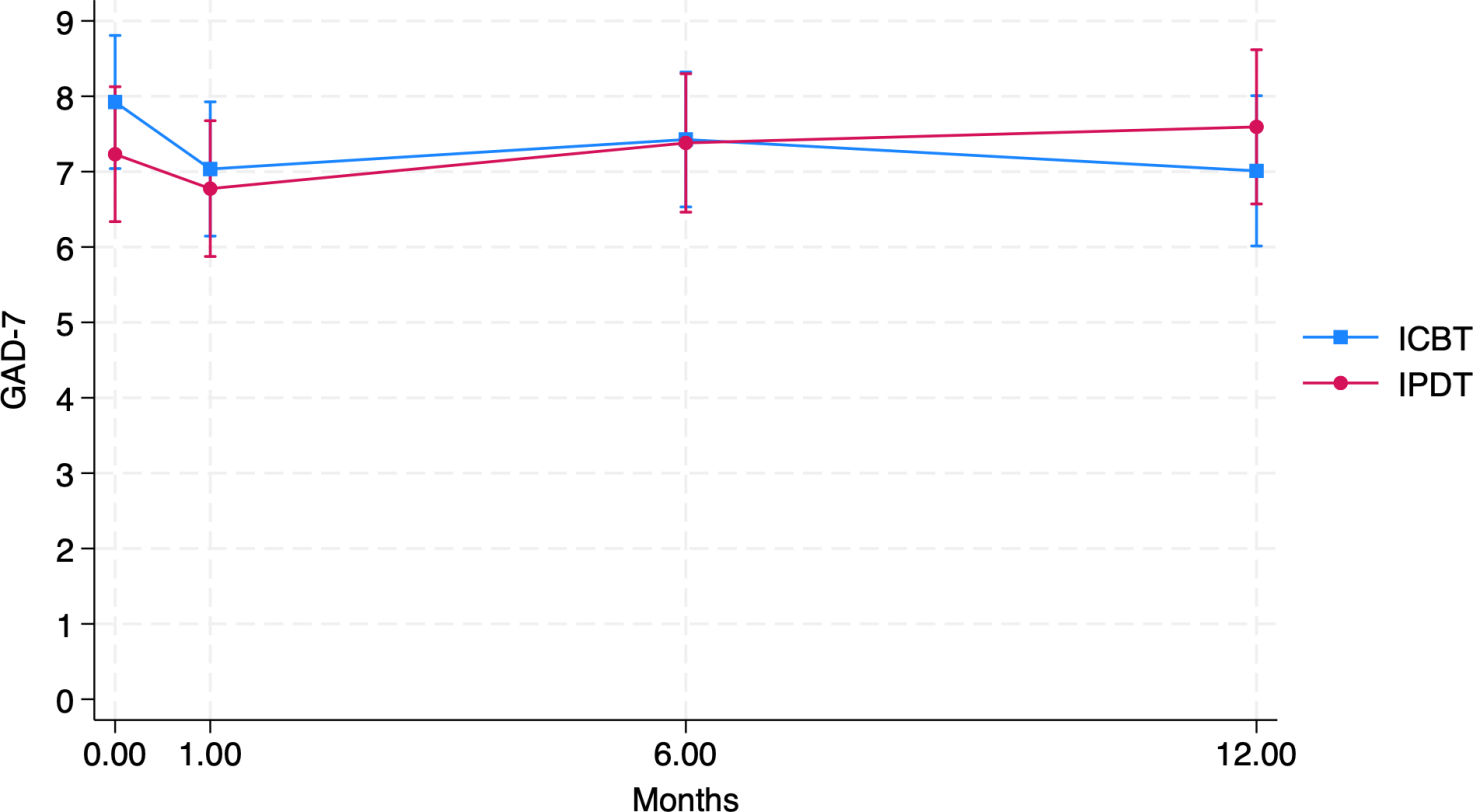
Estimated GAD-7 scores at post-treatment, 1m, 6m and 12m follow-up.

There were no significant changes from treatment endpoint to 12-month follow-up for either group (for ICBT, *p =* 0.052, for IPDT, *p* = 0.456), suggesting that the results were stable during the follow-up, with a trend towards significant further improvement for ICBT. Furthermore, there were no significant interaction effects on any timepoints, suggesting that there were no group differences during the follow-up. The non-significant difference in estimated change between groups in raw scores was 1.28 (95% CI -0.05, 2.60). This corresponds to an effect size of *d =* 0.3 (95% CI -0.01, 0.62). Average anxiety symptom levels were within the mild range (41). Estimated means for each time-point are presented in **Table 2** and illustrated in **Figure 2**.

### Cost-effectiveness

The mean cost of an ICBT treatment was estimated at 21, 479 SEK, corresponding to 2504 USD. The cost of an IPDT treatment was estimated at 20, 972 SEK, corresponding to 2445 USD. However, the cost of any of these treatments with a fully compliant patient is estimated to be approximately 23, 760 SEK.

At the 12-month follow-up, there was no statistically significant difference in healthcare costs between the treatment groups (*p* = 0.324). See **Table 3** for observed medical costs over groups and timepoints. The generalized linear model (GLM) with a gamma distribution and log link, adjusted for baseline medical costs, estimated adjusted mean costs of 13, 177 SEK (1, 536 USD) in the ICBT group and 15, 079 SEK (1, 758 USD) in the IPDT group. The log-scale coefficient for the treatment group was 0.18, corresponding to an approximate 20% higher expected cost in the IPDT group; however, this difference was not statistically significant. Baseline medical costs were a small but significant predictor of 12-month costs (*p* = 0.017), with higher baseline costs associated with slightly higher follow-up costs.

**Table 3:** Observed means (SD) and medians (IQR) in direct medical costs over treatment groups.

	Baseline		12-month follow-up	
Direct medical costs (USD)	ICBT	IPDT	ICBT	IPDT
Mean (SD)	1184.21 (1857.8)	1060.83 (1295.72)	1473.44 (2040.7)	1782.06 (2359.66)
Median (IQR)	582.65 (0 – 1713.32)	564.81 (0-1577.36)	629.64 (0 – 2006.92)	918.57 (0-2459.79)

Medians are calculated on all observed data including zeros.

Sensitivity analyses showed that the results were robust to alternative handling of extreme values and distributional assumptions. Winsorization of healthcare costs at the 1st and 99th percentiles had minimal impact on the results, with adjusted mean costs and group differences changing by less than 3% compared to the primary analysis. In addition, bootstrap resampling with 1, 000 iterations produced confidence intervals that were highly consistent with those obtained from the main GLM model. Together, these findings indicate that the cost comparison was not materially influenced by extreme values or distributional irregularities in the cost data. See **Table 4** for adjusted results.

**Table 4:** Adjusted 12-month healthcare costs by treatment group.

Treatment group	Adjusted mean cost (SEK)	95% CI (SEK)	Adjusted mean cost (USD)	95% CI (USD)
ICBT	23, 287	17, 651 – 31, 450	2, 717	2, 058 – 3, 670
IPDT	27, 760	21, 840 – 35, 406	3, 237	2, 548 – 4, 126

Adjusted mean costs were estimated using a generalized linear model (gamma distribution, log link) controlling for baseline costs. Winsorization of extreme values at the 1st and 99th percentiles and bootstrap resampling (1, 000 iterations) were performed to assess robustness; results were not materially affected.

## Discussion

This study presents follow-up outcomes and a comparative cost analysis of a randomized controlled trial comparing ICBT and IPDT for adolescent depression. The results suggested that treatment gains were stable over time, with no differences between treatments on either symptoms of depression or anxiety. In clinical terms, this stability indicates that the symptom reductions observed at treatment endpoint were largely maintained over the 12-month follow-up period, with no evidence of relapse or substantial symptom worsening at the group level. These findings extend the post-treatment results previously reported by Mechler et al. (18), where both IPDT and ICBT were effective in reducing depressive symptoms immediately following the 10-week intervention, with no differences between the treatments. However, although average symptom levels remained stable, and within the mild range of both depressive symptoms (33) and anxiety symptoms (41), a proportion of participants continued to report clinically relevant symptoms at follow-up, indicating that remission was not universal. It should also be noted that some participants reported receiving additional treatment for depression during the follow-up period, including both talking therapies and pharmacological treatment. Such follow-up care may have contributed to the maintenance of treatment gains for some individuals and should be considered when interpreting the stability of symptom trajectories over time.

There was a non-significant trend toward a group difference on anxiety symptoms during follow-up, where the IPDT group had lower scores at treatment endpoint, but an increase during the follow-up period, while the ICBT group had the opposite trajectory in anxiety symptom levels.

For the assessment of costs and cost-effectiveness, the two treatments were designed to be delivered with comparable costs. Compared to many internet-delivered interventions, both treatments have relatively high levels of delivery cost, due to the 30 minutes of weekly synchronous chat sessions. These chat sessions were added to increase motivation and treatment engagement, which was thought to be especially important for depressed young people. It should be noted that internet-delivered interventions for children and adolescents frequently include synchronous therapist contact elements to a larger extent than comparable programs developed for adults. In the present trial, we included synchronous chat sessions, while other trials have instead included phone calls or videoconferencing (48, 49). Future studies should assess whether the addition of the synchronous chat sessions is related to increased treatment engagement and/or effects in IPDT and/or ICBT. It is likely that chat sessions are important for some patients but not for all. A trial utilizing a factorial design did not find that adding synchronous chat sessions affected outcome in ICBT for anxious youths (50). Earlier studies on IPDT for adults have not included synchronous chat sessions, and treatments have been effective for both depression and anxiety (e.g., 15, 21, 36). Optional chat sessions, chat sessions based on initial assessment, or chat sessions as an addition for patients who are not engaged in treatment are three possible approaches that could be tested in future clinical trials.

The medical cost data, assessed using TIC-P, was highly skewed. The sample included several outliers with very high cost-data, for example one participant in the IPDT group reported 37 visits to medical care during the last three months at the one-year follow-up. Given that the median values are approximately half of the mean values, the cost distribution is clearly right-skewed, with a small number of high-cost cases accounting for a large share of total costs. This is also illustrated in the fact that at follow-up, the 90^th^ percentile is 4254.5 and 4430.3 USD in ICBT and IPDT respectively, while the corresponding numbers for the 99^th^ percentile are more than double, 10374.4 and 10636 USD. It should be noted that the 25 percent quartile is 0 for all groups at both timepoints. However, this is normal for health care cost data, and it would not be appropriate to exclude outliers from the analysis.

The comparison of healthcare costs before treatment and at the 12-month follow up was complicated by the timing of data collection, given that it overlapped with the COVID-19 pandemic and the varying public−health recommendations during that period. Recruitment took place between August 2019 and October 2020, meaning that 12-month follow-ups took place between October 2020 and December 2021. In Sweden, 40 percent of the population states that they refrained from seeking medical care during 2020. Roughly 15 percent state that planned medical care was postponed. In contrast, during 2021, there was a sharp rise in healthcare demand in Sweden (51). The slight increase in healthcare consumption seen from baseline to 12-month follow-up in the present study may therefore reflect a general trend of increased healthcare seeking in the Swedish population. This line of reasoning is supported by another report, showing that the percentage of girls aged 13–17 seeking mental health care in the primary health care system in Sweden increased from around 5.5% in 2019 to over 8% in 2021. For boys, the corresponding change was from around 2.5% to slightly over 3 percent. In specialized mental health care, there was also a slight increase for girls 7–17 during the same period, while boys had a slight decrease in use (52). Considering all this, we chose to report baseline healthcare costs, and adjust for these in the cost-comparison at 12 months, but not analyse pre-post differences.

One major limitation is that there was no control group in the present study, meaning that we cannot control for other systematic influences during the follow-up time. Again, one such possible influence is the COVID-19 pandemic. Participants in the trial were significantly more isolated than usual, with schools closing and directives regarding social distancing during large parts of the follow-up time. It is likely that this has influenced the well-being of the participants, as studies show increase in mental health problems in adolescents during and after the pandemic, compared to before (53–56) and may have affected symptoms during the follow-up. This could potentially add to the non-significant trend of a slight increase in depressive symptoms during this period. However, a meta-analysis on studies of psychotherapy for adolescent depression conducted before the pandemic suggests a similar trend, where effects compared to control groups diminished non-significantly during follow-up (57). Further studies, preferably with a relevant control group, such as treatment as usual, are needed to obtain a clearer view of the sustainability of treatment outcomes in IPDT and ICBT. This would also allow for more sophisticated cost-effectiveness analyses.

A further limitation is the relatively low frequency of assessments during the follow-up period, with intervals of 5–6 months between measurements, compared to weekly assessments during the treatment phase. More frequent follow-up assessments could have increased the robustness of the findings and enabled a higher-resolution characterization of post-treatment symptom trajectories. However, this would increase the burden on participants and likely lead to data attrition. Nevertheless, it is important to note that follow-up assessments are generally scarce in clinical trials involving children and adolescents, which contributes to uncertainty regarding long-term treatment effects (57). A recent meta-analysis emphasized the need for long-term follow-ups of at least one year after treatment termination (26). In this context, the present study contributes to the existing literature by comparing two active treatments with repeated assessments over a 12-month post-treatment follow-up period.

A strength of this study is the high completion-rate on the instruments at follow-up. The completion-rate ranges from 86.8–96% at the different measures and time-points, providing robust estimates.

Future research should incorporate quality of life measures to assess cost utility for example based on Quality of Life Years. In the present study, it was not considered meaningful to calculate ICER for the two treatments since there were almost no differences in effects. With such small nominators, the ICER risks to be inflated (45). Had the present trial included an inactive control group, this would have enabled the calculation of an ICER, allowing for a more precise assessment of the intervention’s relative economic value. Furthermore, even though productivity losses are not relevant for adolescent samples, school attendance could be measured in future trials. Although this is not directly related to societal costs in the short-term, lacking school attendance is highly related to societal costs in the long-term, making this a relevant measure. In addition, future trials should try and estimate work absence related to caregiving. Inclusion of metrics such as school attendance and parental work loss would allow for a more comprehensive societal perspective analysis, capturing the indirect costs that are uniquely significant in paediatric populations (27). Nonetheless, the current focus on direct medical costs provides a robust and necessary foundation for understanding the immediate economic impact on the healthcare system, which is a primary concern for policy makers. Considering that the long-term negative effects of adolescent depression seem to extend into adult life (1, 58, 59), follow-ups in adulthood, such as a 10-year follow-up would be even more interesting.

In conclusion, follow-up analyses found no evidence that any of the treatments outperformed the other in maintaining treatment gains over 12 months, alongside comparable treatment and healthcare costs. These results extend prior post-treatment findings, suggesting both warrant consideration within services—guided by patient preference, therapist expertise, and implementation factors. Longer-term studies with control groups and formal cost-effectiveness evaluations are needed.

**Implications for services:** Given the lack of differences between IPDT and ICBT in maintaining symptom reductions over 12 months, alongside comparable treatment delivery and healthcare costs, service providers can base selection on patient preferences, therapist expertise, and implementation constraints such as training availability and digital infrastructure. Flexible stepped-care models offering both modalities may optimize access and uptake without compromising outcomes. These findings support their joint consideration as pragmatic options for adolescent depression services, pending further cost-effectiveness research.

## Data Availability

The datasets presented in this article are not readily available because participants were mostly minors and the datasets contained sensitive data. In the written informed consent before entering the trial, participants were informed that data from the study, which could not be used to identify them as individuals, could be shared with other researchers. Therefore, the datasets are available if the material requested does not contain information that is classified as secret in accordance with the Public Access to Information and Secrecy Act. The assessment of the information in the material requested must be done at the time of the request and only if the information is secret can the request be denied. Requests to access the datasets should be directed to bjorn.philips@psychology.su.se.

## References

[1] Copeland WE Alaie I Jonsson U Shanahan L . Associations of childhood and adolescent depression with adult psychiatric and functional outcomes. J Am Acad Child Adolesc Psychiatry. (2021) 60:604–11. doi: 10.1016/j.jaac.2020.07.895. PMID: 32758528 PMC8051642

[2] Auerbach RP Mortier P Bruffaerts R Alonso J Benjet C Cuijpers P . WHO World Mental Health Surveys International College Student Project: Prevalence and distribution of mental disorders. J Abnormal Psychol. (2018) 127:623–38. doi: 10.1037/abn0000362. PMID: 30211576 PMC6193834

[3] Collishaw S . Annual research review: secular trends in child and adolescent mental health. J Child Psychol Psychiatry. (2015) 56:370–93. doi: 10.1111/jcpp.12372. PMID: 25496340

[4] Rocha TB-M Graeff-Martins AS Kieling C Rohde LA . Provision of mental healthcare for children and adolescents: a worldwide view. Curr Opin Psychiatry. (2015) 28:330–5. doi: 10.1097/YCO.0000000000000169. PMID: 26001925

[5] Feeney A Hock RS Fava M Hernández Ortiz JM Iovieno N Papakostas GI . Antidepressants in children and adolescents with major depressive disorder and the influence of placebo response: a meta-analysis. J Affect Disord. (2022) 305:55–64. doi: 10.1016/j.jad.2022.02.074. PMID: 35247482

[6] Plöderl M Lyus R Horowitz MA Moncrieff J . The loss of efficacy of fluoxetine in pediatric depression: explanations, lack of acknowledgment, and implications for other treatments. J Clin Epidemiol. (2025) 189:112016. doi: 10.1016/j.jclinepi.2025.112016. PMID: 41548966

[7] Cregeen S Hughes C Midgley N Rhode M Rustin M . Short-term psychoanalytic psychotherapy for adolescents with depression: A treatment manual. 1st. Catty J , editor. London: Routledge (2018). doi: 10.4324/9780429480164, PMID:

[8] McAlpine R Hillin A . Interpersonal psychotherapy for adolescents: a clinician’s guide. Abingdon, Oxon; New York, NY: Routledge (2021).

[9] McCauley E Schloredt KA Gudmundsen GR Martell CR Dimidjian S . Behavioral activation with adolescents: A clinician’s guide. New York: Guilford Publications (2016). 10.1080/15374416.2014.979933 PMC6107350 25602170

[10] Cuijpers P Karyotaki E Ciharova M Miguel C Noma H Furukawa TA . The effects of psychotherapies for depression on response, remission, reliable change, and deterioration: a meta‐analysis. Acta Psychiatrica Scandinavica. (2021) 144:288–99. doi: 10.1111/acps.13335. PMID: 34107050 PMC8457213

[11] Gulliver A Griffiths KM Christensen H . Perceived barriers and facilitators to mental health help-seeking in young people: a systematic review. BMC Psychiatry. (2010) 10:113. doi: 10.1186/1471-244X-10-113. PMID: 21192795 PMC3022639

[12] Andersson G . Internet-delivered CBT: distinctive features. London: Routledge (2025). doi: 10.4324/9781003453444, PMID:

[13] Mamukashvili-Delau M Koburger N Dietrich S Rummel-Kluge C . Long-term efficacy of internet-based cognitive behavioral therapy self-help programs for adults with depression: systematic review and meta-analysis of randomized controlled trials. JMIR Ment Health. (2023) 10:e46925. doi: 10.2196/46925. PMID: 37606990 PMC10481211

[14] Hedman‐Lagerlöf E Carlbring P Svärdman F Riper H Cuijpers P Andersson G . Therapist‐supported internet‐based cognitive behaviour therapy yields similar effects as face‐to‐face therapy for psychiatric and somatic disorders: an updated systematic review and meta‐analysis. World Psychiatry. (2023) 22:305–14. doi: 10.1002/wps.21088. PMID: 37159350 PMC10168168

[15] Johansson R Björklund M Hornborg C Karlsson S Hesser H Ljótsson B . Affect-focused psychodynamic psychotherapy for depression and anxiety through the internet: a randomized controlled trial. PeerJ. (2013) 1:e102. doi: 10.7717/peerj.102. PMID: 23862104 PMC3709106

[16] Johansson R Hesslow T Ljótsson B Jansson A Jonsson L Färdig S . Internet-based affect-focused psychodynamic therapy for social anxiety disorder: a randomized controlled trial with 2-year follow-up. Psychotherapy. (2017) 54:351–60. doi: 10.1037/pst0000147. PMID: 29251954

[17] Lilliengren P Mechler J Lindqvist K Maroti D Johansson R . The efficacy of experiential dynamic therapies: a 10‐year systematic review and meta‐analysis update. Clin Psychol Psychother. (2025) 32:e70086. doi: 10.1002/cpp.70086. PMID: 40411162 PMC12102587

[18] Mechler J Lindqvist K Carlbring P Topooco N Falkenström F Lilliengren P . Therapist-guided internet-based psychodynamic therapy versus cognitive behavioural therapy for adolescent depression in Sweden: a randomised, clinical, non-inferiority trial. Lancet Digital Health. (2022) 4:e594–603. doi: 10.1016/S2589-7500(22)00095-4. PMID: 35803894

[19] Mechler J Lindqvist K Magnusson K Ringström A Krafman JD Alvinzi P . Guided and unguided internet-delivered psychodynamic therapy for social anxiety disorder: a randomized controlled trial. NPJ Ment Health Res. (2024) 3:21. doi: 10.1038/s44184-024-00063-0. PMID: 38730030 PMC11087569

[20] Midgley N Guerrero-Tates B Mortimer R Edbrooke-Childs J Mechler J Lindqvist K . The Depression: Online Therapy Study (D:OTS)—a pilot study of an internet-based psychodynamic treatment for adolescents with low mood in the UK, in the context of the COVID-19 pandemic. Int J Environ Res Public Health. (2021) 18:12993. doi: 10.3390/ijerph182412993. PMID: 34948601 PMC8702018

[21] Johansson R Ekbladh S Hebert A Lindström M Möller S Petitt E . Psychodynamic guided self-help for adult depression through the internet: a randomised controlled trial. PloS One. (2012) 7:e38021. doi: 10.1371/journal.pone.0038021. PMID: 22741027 PMC3362510

[22] Vigerland S Lenhard F Bonnert M Lalouni M Hedman E Ahlen J . Internet-delivered cognitive behavior therapy for children and adolescents: A systematic review and meta-analysis. Clin Psychol Rev. (2016) 50:1–10. doi: 10.1016/j.cpr.2016.09.005. PMID: 27668988

[23] Wu Y Fenfen E Wang Y Xu M Liu S Zhou L . Efficacy of internet-based cognitive-behavioral therapy for depression in adolescents: A systematic review and meta-analysis. Internet Interventions. (2023) 34:100673. doi: 10.1016/j.invent.2023.100673. PMID: 37822787 PMC10562795

[24] Lindqvist K Mechler J Carlbring P Lilliengren P Falkenström F Andersson G . Affect-focused psychodynamic internet-based therapy for adolescent depression: randomized controlled trial. J Med Internet Res. (2020) 22:e18047. doi: 10.2196/18047. PMID: 32224489 PMC7154938

[25] Desai Boström AE Cars T Hellner C Lundberg J . Recovery and recurrence from major depression in adolescence and adulthood. Acta Psychiatrica Scandinavica. (2025) 151:625–33. doi: 10.1111/acps.13785. PMID: 39756801 PMC11962338

[26] Duagi D Carter B Farrelly M Lisk S Shearer J Byford S . Long-term effects of psychosocial interventions for adolescents on depression and anxiety: a systematic review and meta-analysis. eClinicalMedicine. (2024) 68:102382. doi: 10.1016/j.eclinm.2023.102382. PMID: 38273890 PMC10809118

[27] Stanic T Saygin Avsar T Gomes M . Economic evaluations of digital health interventions for children and adolescents: systematic review. J Med Internet Res. (2023) 25:e45958. doi: 10.2196/45958. PMID: 37921844 PMC10656663

[28] Goorden M Van Der Schee E Hendriks VM Hakkaart-van Roijen L . Cost-effectiveness of multidimensional family therapy compared to cognitive behavioral therapy for adolescents with a cannabis use disorder: data from a randomized controlled trial. Drug Alcohol Depend. (2016) 162:154–61. doi: 10.1016/j.drugalcdep.2016.03.004. PMID: 27006273

[29] Goodyer IM Reynolds S Barrett B Byford S Dubicka B Hill J . Cognitive behavioural therapy and short-term psychoanalytical psychotherapy versus a brief psychosocial intervention in adolescents with unipolar major depressive disorder (IMPACT): a multicentre, pragmatic, observer-blind, randomised controlled superiority trial. Lancet Psychiatry. (2017) 4:109–19. doi: 10.1016/S2215-0366(16)30378-9. PMID: 27914903 PMC5285447

[30] Buntrock C . Cost-effectiveness of digital interventions for mental health: current evidence, common misconceptions, and future directions. Front Digital Health. (2024) 6:1486728. doi: 10.3389/fdgth.2024.1486728. PMID: 39498103 PMC11532097

[31] Donker T Blankers M Hedman E Ljótsson B Petrie K Christensen H . Economic evaluations of internet interventions for mental health: a systematic review. Psychol Med. (2015) 45:3357–76. doi: 10.1017/S0033291715001427. PMID: 26235445

[32] Rohrbach PJ Dingemans AE Evers C Van Furth EF Spinhoven P Aardoom JJ . Cost-effectiveness of internet interventions compared with treatment as usual for people with mental disorders: Systematic review and meta-analysis of randomized controlled trials. J Med Internet Res. (2023) 25:e38204. doi: 10.2196/38204, PMID: 36602854 PMC9893732

[33] Bernstein IH Rush AJ Trivedi MH Hughes CW Macleod L Witte BP . Psychometric properties of the Quick Inventory of Depressive Symptomatology in adolescents: QIDS and adolescents. Int J Methods Psychiatr Res. (2010) 19:185–94. doi: 10.1002/mpr.321. PMID: 20683845 PMC2978808

[34] Sheehan DV Lecrubier Y Sheehan KH Amorim P Janavs J Weiller E . The Mini-International Neuropsychiatric Interview (M.I.N.I.): the development and validation of a structured diagnostic psychiatric interview for DSM-IV and ICD-10. J Clin Psychiatry. (1998) 59 Suppl 20:22–33 quiz 34-57. 9881538

[35] Vlaescu G Alasjö A Miloff A Carlbring P Andersson G . Features and functionality of the Iterapi platform for internet-based psychological treatment. Internet Interventions. (2016) 6:107–14. doi: 10.1016/j.invent.2016.09.006. PMID: 30135819 PMC6096305

[36] Mechler J Lindqvist K Philips B Midgley N Lilliengren P . Internet-delivered affect-focused psychodynamic therapy for adolescent depression: treatment principles and clinical application in the ERiCA project. J Infant Child Adolesc Psychother. (2024) 23 (2):123–141. doi: 10.1080/15289168.2024.2339523. PMID: 41858497

[37] Topooco N Berg M Johansson S Liljethörn L Radvogin E Vlaescu G . Chat- and internet-based cognitive-behavioural therapy in treatment of adolescent depression: randomised controlled trial. BJPsych Open. (2018) 4:199–207. doi: 10.1192/bjo.2018.18. PMID: 29988969 PMC6034465

[38] Topooco N Byléhn S Dahlström Nysäter E Holmlund J Lindegaard J Johansson S . Evaluating the efficacy of internet-delivered cognitive behavioral therapy blended with synchronous chat sessions to treat adolescent depression: randomized controlled trial. J Med Internet Res. (2019) 21:e13393. doi: 10.2196/13393. PMID: 31682572 PMC6858617

[39] Kroenke K Spitzer RL Williams JBW . The PHQ-9: validity of a brief depression severity measure. J Gen Internal Med. (2001) 16:606–13. doi: 10.1046/j.1525-1497.2001.016009606.x. PMID: 11556941 PMC1495268

[40] Hlynsson JI Skúlason S Andersson G Carlbring P . Why are we still using the PHQ-9? A historical review and psychometric evaluation of measurement invariance. Psychiatr Q. (2025). doi: 10.1007/s11126-025-10208-9. PMID: 40900414

[41] Spitzer RL Kroenke K Williams JBW Löwe B . A brief measure for assessing generalized anxiety disorder: the GAD-7. Arch Internal Med. (2006) 166:1092. doi: 10.1001/archinte.166.10.1092. PMID: 16717171

[42] Tiirikainen K Haravuori H Ranta K Kaltiala-Heino R Marttunen M . Psychometric properties of the 7-item Generalized Anxiety Disorder Scale (GAD-7) in a large representative sample of Finnish adolescents. Psychiatry Res. (2019) 272:30–5. doi: 10.1016/j.psychres.2018.12.004. PMID: 30579178

[43] Bouwmans C De Jong K Timman R Zijlstra-Vlasveld M Van Der Feltz-Cornelis C Tan SS . Feasibility, reliability and validity of a questionnaire on healthcare consumption and productivity loss in patients with a psychiatric disorder (TiC-P). BMC Health Serv Res. (2013) 13:217. doi: 10.1186/1472-6963-13-217. PMID: 23768141 PMC3694473

[44] Burnham KP Anderson DR . Multimodel inference: understanding AIC and BIC in model selection. Sociological Methods Res. (2004) 33:261–304. doi: 10.1177/0049124104268644. PMID: 41836481

[45] Shields GE Elvidge J . Challenges in synthesising cost-effectiveness estimates. Systematic Rev. (2020) 9:289. doi: 10.1186/s13643-020-01536-x. PMID: 33298168 PMC7727163

[46] Diehr P Yanez D Ash A Hornbrook M Lin DY . Methods for analyzing health care utilization and costs. Annu Rev Public Health. (1999) 20:125–44. doi: 10.1146/annurev.publhealth.20.1.125. PMID: 10352853

[47] Zhou J Williams C Keng MJ Wu R Mihaylova B . Estimating costs associated with disease model states using generalized linear models: A tutorial. PharmacoEconomics. (2024) 42:261–73. doi: 10.1007/s40273-023-01319-x. PMID: 37948040 PMC11424740

[48] Nordh M Wahlund T Jolstedt M Sahlin H Bjureberg J Ahlen J . Therapist-guided internet-delivered cognitive behavioral therapy vs internet-delivered supportive therapy for children and adolescents with social anxiety disorder: a randomized clinical trial. JAMA Psychiatry. (2021) 78:705. doi: 10.1001/jamapsychiatry.2021.0469. PMID: 33978699 PMC8117054

[49] Stjerneklar S Hougaard E McLellan LF Thastum M . A randomized controlled trial examining the efficacy of an internet-based cognitive behavioral therapy program for adolescents with anxiety disorders. PloS One. (2019) 14:e0222485. doi: 10.1371/journal.pone.0222485. PMID: 31532802 PMC6750608

[50] Berg M Rozental A De Brun Mangs J Näsman M Strömberg K Viberg L . The role of learning support and chat-sessions in guided internet-based cognitive behavioral therapy for adolescents with anxiety: a factorial design study. Front Psychiatry. (2020) 11:503. doi: 10.3389/fpsyt.2020.00503. PMID: 32587533 PMC7298729

[51] The Swedish Agency for Health and Care Services Analysis (Myndigheten för Vård- och omsorgsanalys) . Från uppdämt vårdbehov till förlängda köer. In: Uppföljning av förändringar i befolkningens vårdkonsumtion till följd av covid-19-pandemin. The Swedish Agency for Health and Care Services Analysis (Myndigheten för Vård- och omsorgsanalys (2022).

[52] Lalouni M Sakka L Gubi E Dal H Dalman C . Det utökade uppdraget för barn och unga med psykisk ohälsa i Region Stockholm – uppföljning av strukturförändringar inom primärvården. Centrum för epidemiologi och samhällsmedicin, Region Stockholm (2024).

[53] Källmen H Hallgren M . Mental health problems among adolescents during the COVID-19 pandemic: a repeated cross-sectional study from Sweden. Scandinavian J Public Health. (2024) 52:329–35. doi: 10.1177/14034948231219832. PMID: 38217316 PMC11067385

[54] Ludwig-Walz H Dannheim I Pfadenhauer LM Fegert JM Bujard M . Increase of depression among children and adolescents after the onset of the COVID-19 pandemic in Europe: a systematic review and meta-analysis. Child Adolesc Psychiatry Ment Health. (2022) 16:109. doi: 10.1186/s13034-022-00546-y. PMID: 36587221 PMC9805372

[55] Nyberg G Helgadóttir B Kjellenberg K Ekblom Ö . COVID-19 and unfavorable changes in mental health unrelated to changes in physical activity, sedentary time, and health behaviors among Swedish adolescents: a longitudinal study. Front Public Health. (2023) 11:1115789. doi: 10.3389/fpubh.2023.1115789. PMID: 36969680 PMC10036362

[56] Zetterqvist M Landberg Å Jonsson LS Svedin CG . The psychosocial consequences of covid-19 in adolescents with nonsuicidal self-injury. Child Adolesc Psychiatry Ment Health. (2023) 17:33. doi: 10.1186/s13034-023-00566-2. PMID: 36871031 PMC9985473

[57] Eckshtain D Kuppens S Ugueto A Ng MY Vaughn-Coaxum R Corteselli K . Meta-analysis: 13-year follow-up of psychotherapy effects on youth depression. J Am Acad Child Adolesc Psychiatry. (2020) 59:45–63. doi: 10.1016/j.jaac.2019.04.002. PMID: 31004739

[58] Alaie I Ssegonja R Philipson A Von Knorring A-L Möller M Von Knorring L . Adolescent depression, early psychiatric comorbidities, and adulthood welfare burden: a 25-year longitudinal cohort study. Soc Psychiatry Psychiatr Epidemiol. (2021) 56:1993–2004. doi: 10.1007/s00127-021-02056-2. PMID: 33715045 PMC8519903

[59] Alaie I Philipson A Ssegonja R Copeland WE Ramklint M Bohman H . Adolescent depression and adult labor market marginalization: a longitudinal cohort study. Eur Child Adolesc Psychiatry. (2022) 31:1799–813. doi: 10.1007/s00787-021-01825-3. PMID: 34173065 PMC9666342

