# Supplementary material for: Twelve-month outcomes and comparative costs of internet-delivered psychodynamic therapy versus cognitive-behavioral therapy for adolescent depression: a randomized controlled trial
Source: Front Psychiatry. 2026 Apr 20;17:1794684. doi: 10.3389/fpsyt.2026.1794684 (PMC13136967; doi:10.3389/fpsyt.2026.1794684)
Supplement: Supplementary file 1 [file SupplementaryFile1.docx]

Supplemental Table 1.
Therapist time distribution

| **Treatment group** | **Mean number of chats (SD)** | **Mean chat time (SD)** | **Mean weekly feedback time (SD)** | **Total time** |
| --- | --- | --- | --- | --- |
| ICBT | 8.5 (2.81) | 32 min 53 sek (6.1) | 13 min 12 sek (3.50) | 6.78 hours |
| IPDT | 7.85 (3.16) | 33 min 46 sek (5.58) | 13 min 42 sek (3.26) | 6.62 hours |
|  |  |  |  |  |

# Supplemental Table 2. Unit costs and mean units at 12-month follow-up

| **Unit** | **Unit cost (SEK)** | **Mean units ICBT** | **Mean units IPDT** |
| --- | --- | --- | --- |
| General practitioner | 2728 | 1.15 (1.86) | 1.30 (2.09) |
| Nurse | 1772 | 0.79 (1.54) | 0.87 (1.53) |
| Counseller/Social worker | 2422 | 0.74 (1.91) | 1.0 (2.5) |
| Physiotherapist/logoped/dietist | 1970 | 0.38 (1.34) | 0.31 (1.25) |
| Psychologist, psychotherapist, psychiatrist | 3320 | 0.89 (2.19) | 1.22 (2.28) |
| Other staff within mental healthcare | 2700 | 0.13 (0.60) | 0.15 (0.59) |
| Alcohol/drug care | 2214 | 0.05 (0.471) | 0.00 (0.00) |
| Emergency care | 6300 | 0.12 (0.38) | 0.12 (0.35) |
| Inpatient care (any) | 9151 | 0.02 (0.26) | 0.03 (0.21) |
| Specialist practitioner | 6454 | 0.18 (0.58) | 0.26 (0.75) |
